# [Retracted] MicroRNA-216b-3p inhibits lung adenocarcinoma cell growth via regulating PDZ binding kinase/T-LAK-cell-originated protein kinase

**DOI:** 10.3892/etm.2025.13047

**Published:** 2025-12-15

**Authors:** Yaqin Chai, Huijun Xue, Yanmei Wu, Xiaomei Du, Zhuohong Zhang, Yinliang Zhang, Lili Zhang, Shuanbao Zhang, Zhiguo Zhang, Zhiwen Xue

Exp Ther Med 15:4822–4828, 2018; DOI: 10.3892/etm.2018.6020

Following the publication of this paper, it was drawn to the Editor's attention by a concerned reader that areas of the flow cytometric (FCM) plots featured in Fig. 5A on p. 4826 were strikingly similar to FCM data which were ultimately published in a number of other papers in different journals that were written by different authors at different research institutes, including an article that was submitted at around the same time as this one to the journal *Cancer Biomarkers,* which has since been retracted.

Owing to the fact that the contentious data in the above article were found to be strikingly similar to data that have appeared elsewhere in other papers in the scientific literature, the Editor of *Experimental and Therapeutic Medicine* has decided that this paper should be retracted from the Journal. The authors were asked for an explanation to account for these concerns, but the Editorial Office did not receive a reply. The Editor apologizes to the readership for any inconvenience caused.

